# Addressing Selection Bias When Estimating Associations Between Physical and Cognitive Functioning in Older Adult Women

**DOI:** 10.1002/alz70860_107176

**Published:** 2025-12-23

**Authors:** Lauren P. MacConnachie, Jillian S. Baker, Michelle M. Hood, Alexis Reeves, Carol A. Derby, Kelly R. Ylitalo, Aleda M. Leis, Kelley Pettee Gabriel, Sherri‐Ann M. Burnett‐Bowie, Bradley M. Appelhans, Carrie A. Karvonen‐Gutierrez

**Affiliations:** ^1^ University of Michigan, Ann Arbor, MI, USA; ^2^ Stanford University, Stanford, CA, USA; ^3^ Department of Neurology, and Department of Epidemiology and Population Health, Albert Einstein College of Medicine, Bronx, NY, USA; ^4^ Baylor University, Waco, TX, USA; ^5^ The University of Alabama at Birmingham, Birmingham, AL, USA; ^6^ Harvard Medical School, Boston, MA, USA; ^7^ Rush University, Chicago, IL, USA

## Abstract

**Background:**

Physical functioning is positively associated with cognitive performance but estimates of this association from longitudinal studies may be subject to selection bias due to differential loss‐to‐follow‐up via poor physical function, death, or comorbidities. We estimated the potential impact and drivers of differential selection on this association among a cohort of older women.

**Method:**

The Study of Women's Health Across the Nation (SWAN) is a community‐based, longitudinal study of women. From the initial cohort (*n* = 3,302), only 59.4% (*n* = 1345) participants completed gait speed and cognitive functioning tests at study visit 15 (V15, 2015‐16). To address potential selection bias for each of the reasons above, inverse probability weighting was used to upweight participants at V15 who resembled those lost to follow‐up.

A global cognitive z‐score was derived by averaging z‐scores from processing speed, verbal memory, and working memory tests; higher z‐score indicates better overall cognitive functioning. Linear regression models (with and without weights) estimated the relationship between gait speed and global cognitive z‐score. Models were stratified by financial strain and education.

**Result:**

Our sample included 1345 women with a mean age of 65.5 (± 2.6 SD) years.

The unweighted, unstratified model estimated a faster gait speed was associated with a 0.82‐unit increase in global z‐score (95% CI:0.66,0.98). The weighted model estimate was 17% higher (β:0.96, 95%CI:0.77,1.15).

Among women who reported any financial strain, the unweighted model found that a faster gait speed was associated with a 1.08‐unit increase in global z‐score (β:1.08, 95%CI:0.74,1.42), whereas the weighted model estimated this association to be 13.9% higher (β:1.23, 95%CI: 0.86,1.60).

Comparatively, among women who reported no financial strain, the weighted model estimate was 6.8% higher than the unweighted model (weighted β:0.63, 95%CI:0.44,0.83; unweighted β:0.59, 95%CI:0.42,0.77). Similar results were observed in models stratified by education.

**Conclusion:**

Results indicate that loss to follow‐up in SWAN leads to underestimation of the relationship between gait speed and cognitive performance. The association is strongest among the most disadvantaged, who are also more likely to drop out. Accounting for differential loss‐to‐follow‐up is critical for a more accurate characterization of the association between gait speed and cognitive performance.

